# Kidney Paired Exchange: a step too far or a winning hand?

**DOI:** 10.1590/2175-8239-JBN-2021-0272

**Published:** 2022-02-21

**Authors:** Song C Ong, Vineeta Kumar

**Affiliations:** 1 University of Alabama, Birmingham, AL, USA.

Kidney transplantation, whether from a deceased or living donor, improves the quality of life and survival of patients with end-stage kidney disease (ESKD) and advanced chronic kidney disease (CKD)^1^.The demand for organs far exceeds supply, and waitlists for deceased donor organ is growing worldwide. For many people, the wait for a life-saving transplant can stretch over many years and never become a reality. Recipients of allografts from living donors have significant advantages over those who receive deceased-donor grafts in terms of graft and patient survival. In this context, one in three potential living kidney donors is ABO-incompatible or crossmatch positive with the intended recipient^2^. Such donors have been traditionally deemed “incompatible,” irrespective of their motivation to donate and their health status, limiting the potential donor pool. Kidney paired donation (KPD) is a potential way to overcome this barrier for this group of donor-recipient pairs^2^. The first paired kidney exchange was performed in 1991 and has since gained popularity all over the world^3^. In its simplest form, KPD involves the simultaneous exchange of organs between two sets of incompatible donor-recipient pairs, and with the growth of registries and collaboration between centers, more complex donor-recipient chains have developed and now even include international donations. All of this is accomplished with excellent donor and recipient outcomes, with patient and graft outcomes after KPD at least comparable to, and probably better than, those after standard living-donor kidney transplantation^4^.

In spite of the growing popularity of KPD, this practice has not yet caught on in South America. In this issue of the Brazilian Journal of Nephrology, Medina-Pestana et al.^5^ and Bastos et al.^6^ debate the merits and potential pitfalls of KPD. The authors of both articles summarize the literature in the field of KPD, with Bastos et al.^6^ coming out strongly in favor of KPD, while Medina-Pestana et al.^5^ express great caution not only with KPD but also with living donation and perhaps transplantation in general.

Bastos et al.^6^ cite the positive impact of KPD in facilitating kidney transplantation, especially for patients who are disadvantaged by their blood type or sensitization history. This is supported by published outcomes such as those from the National Kidney Registry (NKR) in the US^4^. KPD has allowed more African American patients, who are historically over-represented on waitlists and who are more often of blood type B, and patients who are highly sensitized, being able to receive a transplant. The results in these higher-risk populations compared favorably with those of non-KPD living-donors, although cold ischemia time and incidence of delayed graft function increase in KPD chains that involve transportation of a living donor organ. They acknowledge that despite its bureaucratic complexity, requiring matching algorithms, organ shipping, and coordination between transplant centers, KPD has been successfully performed in multiple countries, both developed and under developed. Little to no negative political or social consequences have been noted, demonstrating that KPD is a valuable resource for further improving the quality of life and survival of people with end-stage kidney disease.

Despite the growing body of literature, concerns about the practice of KPD remain, and as recently as 2018, the Brazilian Federal Council of Medicine urged caution. It raised concerns that KPD may result in worse outcomes, benefit only a minority in a country with large social-economic disparities, and a negative impact on transplantation rates in the country.

Medina-Pestana et al.^5^ raise several concerns, including the fear that only the wealthy will benefit and that devoting resources to promote KPD would divert attention from the deceased donor program. The authors are concerned that living donation poses potential risks to the long-term health of the donor, which may be exacerbated by a lack of medical care, especially in resource-poor countries^7^ and therefore advocate for a cautious approach to living kidney donation in general. While donor risk may be ethically justified, they fear that the link between donor and recipient will be diluted in a KPD as all donor-recipient pairs are unrelated. They worry that poor outcomes of KPD might adversely affect organ donation. In conclusion, Medina-Pestana et al.^5^ urge caution in blindly assuming that renal transplantation is superior to dialysis, especially as patients are at risk for immunosuppression and for disease transmission with organ transplantation^8^.

The ethics of KPD are complex^9^. Intense and lively discourse is crucial, as each society must decide for itself how to prioritize resources for the care of the sick and disadvantaged. Furthermore, with regard to living organ donors, they must decide how best to protect their interests and long-term health. By bringing awareness of KPD to the medical community in Brazil, both Medina-Pestana et al.^5^ and Bastos et al.^6^ and colleagues are furthering the cause of transplantation in their community. And if one is convinced that kidney transplantation is a life-enhancing and life-extending therapy, then KPD should be considered as an option for all transplant candidates who have a living donor who is medically able but cannot donate a kidney to the intended candidate because of ABO or human leukocyte antigen (HLA) incompatibility.
